# Acute myocardial infarction following intravenous tissue plasminogen activator for acute ischemic stroke: An unknown danger

**DOI:** 10.4103/0972-2327.61282

**Published:** 2010

**Authors:** Adatia Sweta, Sanghani Sejal, Sanzgiri Prakash, Chauhan Vinay, Hastak Shirish

**Affiliations:** Department of Neurology, Lilavati Hospital and Research Center, Bandra (West), Mumbai-400 050, India; 1 Department of Cardiology, Lilavati Hospital and Research Center, Bandra (West), Mumbai-400 050, India

**Keywords:** Acute myocardial infarction, acute ischemic stroke, intravenous tissue plasminogen activator

## Abstract

Thrombolysis with intravenous tissue (IV) plasminogen activator (tPA) is considered for patients with acute ischemic stroke falling within the described inclusion criteria defined by The National Institute of Neurological Disorders and Stroke (NINDS) rtPA trial. Complications of IV thrombolysis with tPA are commonly related to hemorrhage, anaphylaxis, or arterial occlusion. We describe two cases of acute myocardial infarction (MI) following IV tPA infusion for acute stroke. One of the patients had underlying ischemic heart disease (IHD) while the other did not have any prior IHD. Both had presented with acute ischemic stroke within the window period of thrombolysis and had no contraindications for thrombolysis. Both the patients succumbed due to myocardial infarction and cardiovascular collapse due to new onset arrhythmias. Acute MI immediately following IV tPA for stroke is a rare but serious complication. The disruption of intracardiac thrombus and subsequent embolization to coronary arteries may be an important mechanism in the occurrence of MI after administration of tPA for acute ischemic stroke. As both the patients succumbed before the arrangement for coronary angiography, the demonstration of intracardiac or intracoronary thrombus was not possible. But clinically, the presence of chest pain with elevated troponin levels and ST segment elevation pointed to MI. We suspect that fragmentation and lysis of intracardiac thrombus may result in MI after use of tPA for acute ischemic stroke, though the remote possibility of simultaneous occurrence of two atherosclerotic events MI and stroke exists.

## Introduction

Thrombolysis with intravenous tissue (IV) plasminogen activator (tPA) is considered for patients with acute ischemic stroke falling within the described inclusion criteria defined by The National Institute of Neurological Disorders and Stroke (NINDS) rtPA trial.[1] The complications of this therapy, as we know till date, are limited to hemorrhage and allergic reactions.[2] However, the spectrum of complications seems to be expanding with unknown factors being discovered with experience. The reports of development of acute myocardial infarction following administration of IV tPA in acute ischemic stroke have recently been recognized.[3,4] We too came across two such cases over a period of 15 days, these patients developed MI following IV tPA and finally succumbed to death. One of the patients had past history of ischemic heart disease (IHD), while the other had no history of the same. These cases are presented to highlight the need of studying further the corelation between the occurrence of MI following the administration of IV tPA in the management of acute stroke.

## Case Reports

### Case 1

A 78-year-old right-handed male presented to the emergency department with sudden onset of left sided weakness with a past history of hypertension. He had left hemineglect, right gaze preference and left hemiparesis. CT scan revealed no hemorrhage. Baseline vitals were stable with pulse of 84/min regular in rhythm and blood pressure 160/100 mm Hg. His baseline ECG showed left ventricular hypertrophy (LVH) with no evidence of ischemia. He was diagnosed as right middle cerebral artery (MCA) stroke syndrome with unclear mechanism and administered IV tPA in usual dosage (0.9 mg/ kg) at 160 minutes following the onset of stroke.

Two hours following the infusion of tPA, he developed chest pain and a while later developed sudden breathlessness. His blood pressure dropped to 90/60 mm Hg. His pulse rate was 74/min and regular. His ECG showed an acute inferolateral wall myocardial infarction. Troponin-T was sent immediately.

His echocardiography revealed an ejection fraction (EF) of 20% with lateral wall hypokinesia. There were no clots/vegetations. CT brain scan was done to rule out any chance of bleeding in the brain. There was no hemorrhage in the CT scan.

The troponin-T level was 2.15. The patient was offered aspirin and the hemodynamic parameters were taken care by adequate inotropic support. Also, urgent Cardiologist's opinion was called for and options for management of MI was sought. The arrangement for coronary angiography was made, however, in the mean time the patient succumbed due to development of ventricular fibrillation.

### Case 2

A 58-year-old right-handed woman with a history of diabetes mellitus (DM), hypertension, IHD, and chronic kidney disease, on maintenance dialysis presented to the ICU from the dialysis unit when the doctors found her developing sudden onset of right-sided weakness after the completion of the dialysis. She was immediately assessed by a neurologist in the ICU, where she was found to have right-sided hemiplegia with right upper motor neuron facial palsy with dysarthria. Her pulse and blood pressure were stable. She was shifted for the CT brain scan immediately which revealed a hyperdense left middle cerebral artery (MCA) with old bilateral basal ganglia lacunar infarcts. She was diagnosed to have acute left MCA stroke with unknown mechanism and received IV tPA 80 minutes after the stroke.

Following the infusion of IV tPA, the power of left arm and leg improved.Approximately two hours after the completion of tPA,she developed sudden onset of chest pain with cardiovascular collapse. Her blood pressure was 60 mm Hg systolic with pulse rate of 30/min. Immediately, she was started on inotropes. ECG revealed ST segment elevation in anterior leads. Intravenous atropine was started. A temporary pacemaker was inserted. Her troponin-T was sent which was elevated to 7.63. Echocardiogram revealed an ejection fraction of 15% with hypokinetic anterior and lateral wall. There was no cardiac thrombus.

Her baseline ECG was reassessed which showed QS pattern in II, III, aVF indicating an old inferior wall myocardial infarction. Neurological examination revealed that she was unresponsive to verbal commands. Pupils were dilated bilaterally (3 mm) in size and reactive. She had a withdrawal response in her left arm and leg. The mechanism of myocardial injury was postulated to be thrombolytic mediated distal embolization of the proximal thrombus in the coronary arteries. Again the patient succumbed before the arrangement for coronary angiography could be made.

## Discussion

We report two cases of myocardial infarction occurring immediately following infusion of IV tPA for acute ischemic stroke. We suspect that systemic thrombolysis with IV tPA contributed to the fragmentation and lyses of underlying cardiac thrombus which subsequently embolized distally to block the coronary circulation and cause myocardial infarction.

There are cases in literature demonstrating the reverse phenomena of development of acute ischemic stroke after administration of thrombolytic therapy in MI.[5] There are postulations in the literature regarding the stroke contributing to the disruption of cardiovascular physiology and causes contributing to the concurrent development of stroke and MI like aortic dissection, large vessel arteritis, endocarditis and drugs like amphetamine and cocaine.[3]

These etiologies were not found in our patients. A stroke involving the insular cortex is known to cause arrhythmias and death.[6] This is thought to be secondary to the role of insular cortex in the autonomic regulation. However in both the patients the presence of chest pain and a clear rise of Troponin-T with elevation of ST segments points clinically to the occurrence of myocardial infarction. Changes in ECG like ST depression, atrial fibrillation and elevated troponin-T is known to occur with acute ischemic stroke but there is no associated pain in the chest.[7]

The development of MI related to the use of IV tPA in our case report is likely as both the myocardial infarcts occurred within 24 hours after receiving the IV tPA and none of the patients had signs and symptoms of impending MI on admission. Moreover, case 1 had no history suggestive of IHD. The simultaneous occurrence of two atherosclerotic events MI and stroke independent of each other is a possibility but such occurrence is very rare.

Cardiac thrombus in the presence of acute ischemic stroke is a relatively rare occurrence. In one of the studies it was found that the cardiac thrombus was present in 2.7% of patients given IV tPA for acute ischemic stroke using transesophageal echocardiography (TEE). In the patients, who did have a cardiac thrombus identified, no patients had evidence of early systemic, coronary or cerebral embolization following the use of IV tPA. However, one patient did develop late recurrent cerebral embolization.[8]

Another study, however, has detected cardiac thrombus in up to 26% of consecutive patients admitted for work up of transient ischemic attack (TIA) or ischemic stroke using TEE.[9] However, the incidence of embolization following the use of IV tPA is not studied by this group.

In conclusion, these cases bring to our notice that the administration of IV tPA may lead to fragmentation and lyses of intracardiac thrombus with subsequent embolization to coronary arteries leading to MI. The clear clinical presentation of MI led us to assume the above mechanism as coronary angiography was not feasible in both the cases and hence documentation of thrombus was not possible.

The management of MI in the event of tPA administered recent stroke like in both our cases is very difficult and no guidelines exist at present for the time duration within which antiplatelets or anticoagulants can be safely started to prevent the cerebral hemorrhage. However, in cases of impending cardiovascular failure, urgent primary coronary angioplasty is justified.[2]
